# Prediction of long-term survival in gastric cancer patients after immunotherapy based on CT-derived extracellular volume fraction

**DOI:** 10.3389/fonc.2025.1698065

**Published:** 2025-11-28

**Authors:** Yuyang Wang, Shanshan Jiang, Yanjie Yang, Yi Li, Zhiying Li, Ziqiao Lei, Xiangchuang Kong, Guofeng Zhou

**Affiliations:** 1 Department of Radiology, Union Hospital, Tongji Medical College, Huazhong University of Science and Technology, Wuhan, China; 2 Hubei Provincial Clinical Research Center for Precision Radiology and Interventional Medicine, Wuhan, China; 3 Hubei Key Laboratory of Molecular Imaging, Wuhan, China; 4 Department of Gastrointestinal Surgery, Union Hospital, Tongji Medical College, Huazhong University of Science and Technology, Wuhan, China

**Keywords:** gastric cancer, immunotherapy, PD-1 inhibitors, biomarkers, extracellular volume fraction, extracellular matrix, contrast-enhanced CT

## Abstract

**Background:**

Gastric cancer remains a leading cause of cancer-related mortality. While immune checkpoint inhibitors (ICIs) have emerged as promising therapies, their efficacy is hindered by the lack of robust patient-centric biomarkers. CT-derived extracellular volume fraction (ECV) has emerged as a novel approach for non-invasive quantification of the extracellular matrix (ECM). This study assesses the predictive value of ECV, a non-invasive imaging biomarker, in gastric cancer patients receiving programmed death receptor-1 (PD-1) inhibitors.

**Methods:**

A retrospective study was conducted on 101 gastric adenocarcinoma patients (stage III: *n* = 47; stage IV: *n* = 54) treated with PD-1 inhibitors at Wuhan Union Hospital from June 21, 2020 to January 3, 2024. The patients were stratified into high- and low-ECV groups using X-tile software. Survival outcomes were compared using Kaplan–Meier curves and log-rank tests. Cox regression analyses identified independent prognostic factors. Two predictive models were developed and evaluated via receiver operating characteristic (ROC) curves and area under the curve (AUC), with internal validation using 1,000 bootstrap iterations.

**Results:**

Kaplan–Meier survival curves indicated that the ECV-higher group had shorter progression-free survival (PFS) (*P* < 0.001) and overall survival (OS) (*P* < 0.001) than the ECV-lower group. Multivariate Cox regression analysis confirmed that high CT-ECV was independently associated with worse PFS and OS (PFS: HR = 2.716, 95% CI: 1.432–5.152, *P* = 0.002 and OS: HR = 2.593, 95% CI: 1.322–5.084, *P* = 0.006).

**Conclusion:**

CT-derived ECV may serve as an independent predictor of long-term survival in gastric cancer patients undergoing immunotherapy.

## Introduction

Gastric cancer is the fifth most common malignant tumor worldwide, with the 2024 statistics showing over 960,000 new cases annually and its ranking as the fifth leading cause of cancer-related deaths (1). In recent years, immune checkpoint inhibitors (ICIs), such as programmed cell death ligand-1 (PD-L1)/programmed death receptor-1 (PD-1) inhibitors, have significantly improved the survival outcomes for some advanced gastric cancer patients. However, existing biomarkers like PD-L1 expression, microsatellite instability (MSI), and Epstein–Barr virus (EBV) infection status, while partially predictive of therapeutic efficacy, exhibit high heterogeneity in expression influenced by tumor histotype, individual genetic background, and dynamic microenvironment changes (2). This results in most patients failing to benefit from immunotherapy, with some potentially experiencing worsened conditions due to immune-related adverse events (irAEs). Therefore, identifying novel biomarkers that overcome heterogeneity limitations has become an urgent need to optimize precision stratification in gastric cancer immunotherapy.

The extracellular matrix (ECM), a three-dimensional macromolecular network composed of collagens, proteoglycans/glycosaminoglycans, elastin, fibronectin, laminins, several other glycoproteins, and various molecules (cytokines, growth factors, and hormones) (3), is essential for cellular functions including proliferation, migration, and differentiation through cell–ECM interactions (4). As a dynamic structure present in all tissues, ECM not only provides structural integrity but also maintains homeostasis through continuous remodeling in response to environmental stimuli (5, 6). Dysregulated ECM remodeling accelerates disease progression and tumor-associated ECM alterations promote tumor growth through tumor-stromal signaling, while increased matrix stiffness facilitates tumor invasion. This vicious cycle creates dense ECM barriers around tumor cells that physically impede drug penetration, reducing therapeutic efficacy (7, 8).

Traditional ECM assessment relying on invasive biopsy staining techniques (e.g., Masson staining) suffers from limitations in dynamic monitoring. Recently, radiomics-based extracellular volume fraction (ECV) technology has emerged as a novel approach for noninvasive ECM quantification. By calculating the distribution differences of intravenous contrast agents (e.g., iodinated contrast) in extracellular spaces, ECV indirectly reflects the ECM fibrosis degree and spatial distribution (9). Originally developed for cardiac fibrosis assessment (10), ECV has demonstrated value in evaluating liver cirrhosis and pancreatic fibrosis and predicting tumor regression grade after neoadjuvant therapy in gastric cancer and postoperative pancreatic complications (11–13). Multiple studies have validated ECV as a quantitative biomarker for ECM assessment in various solid tumors (14–18). Although ECV’s potential in gastric cancer has gained attention (4, 19–23), its clinical translational value for predicting immunotherapy outcomes remains unclear. This study proposes using contrast-enhanced computed tomography (CECT) to measure ECV, aiming to quantify gastric cancer ECM fibrosis as a reflection of suppressive tumor immune microenvironment status, thereby predicting survival benefits from PD-1/PD-L1 inhibitor therapy.

## Methods

### Patients

A total of 195 patients with gastric adenocarcinoma who received PD-1 inhibitor immunotherapy at Wuhan Union Hospital between June 21, 2020 and January 3, 2024 were initially enrolled. All enrolled patients were treated with anti-PD-1 immunotherapy. The vast majority received it in combination with standard first-line chemotherapy. The anti-PD-1 inhibitors used in this cohort included nivolumab, pembrolizumab, sintilimab, tislelizumab, camrelizumab, toripalimab, and envafolimab. The chemotherapy regimens consisted of XELOX (capecitabine and oxaliplatin), SOX (S-1 and oxaliplatin), or FOLFOX (leucovorin, fluorouracil, and oxaliplatin). The choice of therapeutic regimen was determined by the treating oncologist based on contemporary clinical guidelines and the patient’s individual condition. The exclusion criteria were as follows: (a) prior or planned curative-intent gastrectomy, (b) prior neoadjuvant therapy before surgery, (c) iodine contrast allergy, (d) inadequate gastrointestinal preparation leading to unclear tumor visualization, and (e) absent or poor contrast enhancement. Ultimately, 101 patients who underwent CECT before immunotherapy were included for analysis. Clinical and biochemical data were collected retrospectively. These included age, sex, hematocrit levels, and serum tumor markers, specifically carbohydrate antigen 19-9 (CA19-9) and carcinoembryonic antigen (CEA). Laboratory values were obtained from routine blood tests performed within 7 days of the CT scan. The study adhered to the principles of the Declaration of Helsinki and was approved by the Medical Ethics Committee of Wuhan Union Hospital. Written informed consent was waived due to the retrospective nature of the study.

### Image acquisition

All patients provided written informed consent for abdominal CECT. Before scanning, an 8-h fasting period and iodine allergy screening were required. Abdominal imaging was performed using a 128-detector-row CT scanner (Siemens SOMATOM Definition AS+, Erlangen, Germany) with standardized parameters: tube voltage of 120 kV, automated tube current modulation, 1.5 mm slice thickness and interval, and 512 × 512 acquisition matrix. The patients were positioned supine, and nonionic iodinated contrast (350 mgI/mL) was administered via the antecubital vein at a flow rate of 2 to 3 mL/s. Contrast-enhanced images were acquired during the arterial phase (25–30 s post-injection), portal venous phase (50–60 s), and equilibrium phase (acquired at a fixed delay of 180 s post-injection). A research study has revealed that when estimating the ECV, the equilibrium phase at 180 s represents a good balance between the clinical workflow and technical success (24).

### Image analysis

**Figure 1:**
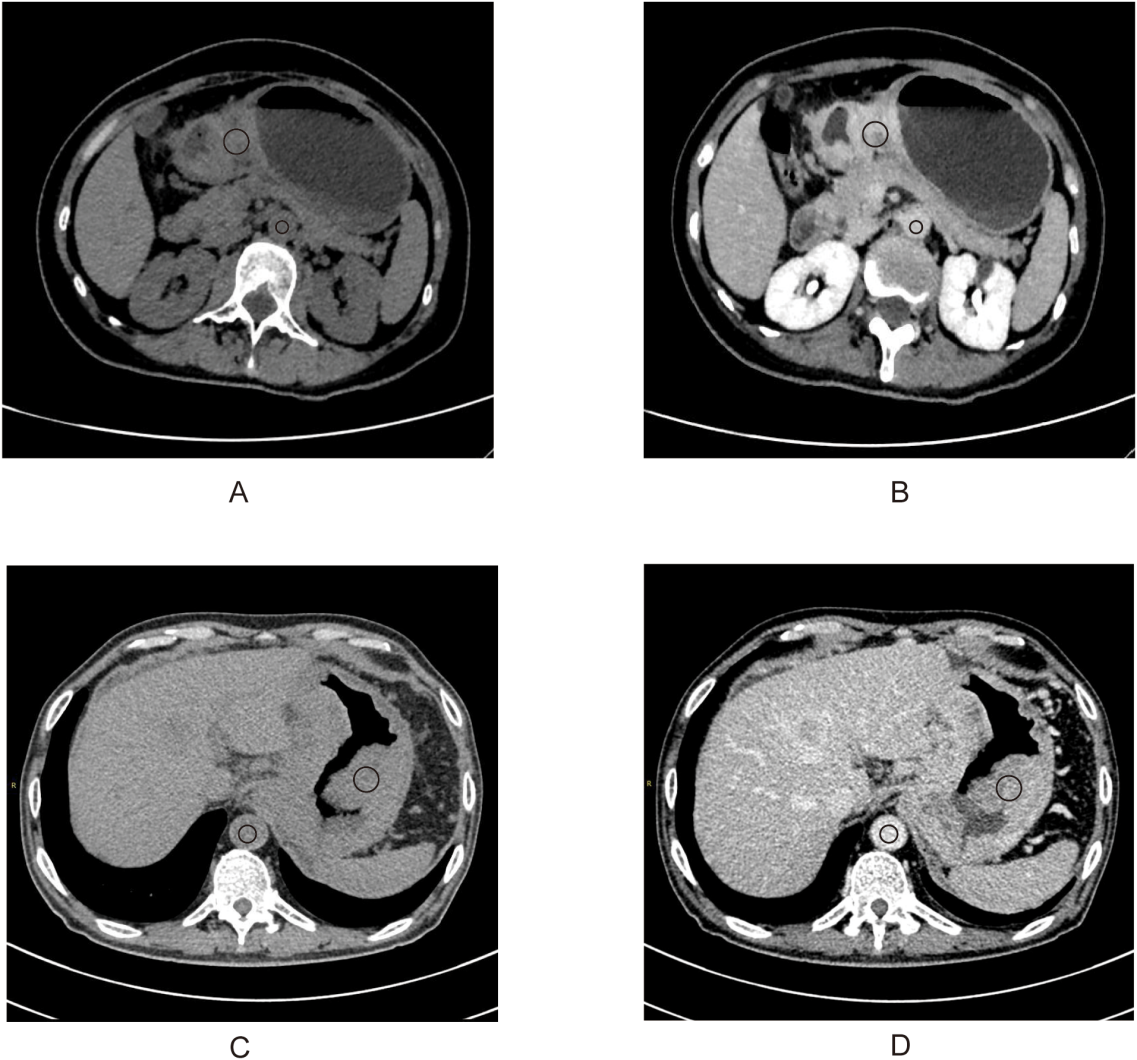
A 50-year-old woman with stage III gastric adenocarcinoma **(A, B)** and a 63-year-old man with stage IV gastric adenocarcinoma **(C**, **D)**. Axial unenhanced **(A**, **C)**; equilibrium-phase contrast-enhanced **(B**, **D)**. The tumor extracellular volume fractions were 31.8% and 35.8%, respectively.


Two radiologists blinded to clinicopathological data (except tumor location) independently measured attenuation values on unenhanced CT and equilibrium-phase CECT. Regions of interest (ROIs) were manually placed within the tumor and the aorta at the same anatomical level (**Figure 1**). The extracellular volume fraction (ECV) was calculated using the following formula:

ECV(%)=(1−hematocrit)×(ΔHUtumor/ΔHUaorta)×100

where ΔHUtumor and ΔHUaorta are the HU in the equilibrium phase minus the HU before the contrast agent administration of the tumor and the aorta, respectively (20). The final ECV value used for analysis was the average of the two radiologists’ measurements.

### Endpoints of the study

The primary endpoint was overall survival (OS), defined as the time from the start of immunotherapy to death or the end of follow-up in cancer patients. The secondary endpoint was progression-free survival (PFS), defined as the time from the start of treatment to disease progression or death from any cause.

### Statistical analysis

To evaluate the consistency of ECV measurements between two radiologists, we employed Bland–Altman analysis and the intraclass correlation coefficient (ICC). An ICC >0.90 was considered indicative of excellent consistency (25). Using X-tile (https://medicine.yale.edu/lab/rimm/research/software/) to get the optimal cutoff values (33.1), the patients were divided into high- and low-ECV groups. X-tile employs an enumeration method that systematically tests all possible values of the continuous variable as candidate cutoff thresholds. A log-rank test was subsequently applied to compare survival curves between the stratified groups, and the cutoff point yielding the smallest *p*-value was selected as the optimal threshold (26). Normally distributed continuous variables were compared using Student’s *t*-test and expressed as mean ± standard deviation (SD). Categorical variables were analyzed via chi-square or Fisher’s exact tests and reported as percentages (*n*, %). The PFS and OS between the high- and low-ECV groups were compared using log-rank test and visualized with Kaplan–Meier curves. Univariate and multivariate Cox regression analyses identified prognostic factors, with variables showing *p* < 0.05 in univariate analysis included in the multivariate model. The results were reported as hazard ratios (HRs) with 95% confidence intervals (CIs). Subgroup analyses were conducted to explore the consistency of the ECV effect. Two predictive models were constructed: model 1 included clinical stage, age group, and sex, while model 2 encompassed all variables from model 1 plus ECV stratification. The predictive performance was evaluated using ROC curves and AUC. Internal validation of AUC values was conducted using a bootstrap resampling method with 1,000 iterations. Statistical significance was defined as *p* < 0.05. Analyses were performed using SPSS 27.0 (IBM Corp.) and R 4.4.2 (R Foundation for Statistical Computing).

## Results

### Interobserver agreement of ECV measurements

The Bland–Altman analysis demonstrated excellent agreement between two radiologists in CT-ECV quantification (**Supplementary Figure S1**). The mean bias was 0.82 units (95% CI: 0.12–1.53), with 95% limits of agreement (LoA) ranging from -6.20 to +7.85. Approximately 95% of data points fell within the LoA, confirming high measurement reproducibility. This was further supported by an ICC of 0.971 (95% CI: 0.957–0.981) for average measures, indicating good consistency.

### Patients’ baseline characteristics

A total of 101 gastric adenocarcinoma patients were included in this study. The average age was 59.3 ± 11.9 years, with 66% being male. Based on the ECV indices and using X-tile, the optimal cutoff values were determined (33.1). There were 48 patients in the low-ECV group (<33.1) and 53 patients in the high-ECV group (≥33.1). No significant differences were observed in age, sex distribution, clinical stage, tumor location, tumor markers, HCT, BMI, risk factors, or other comorbidities (**Table 1**).

**Table 1:** Baseline characteristics of patients.

Characteristics	Low-ECV group (<33.1)	High-ECV group (≥33.1)	*P*-value
Patients, *n*	48	53	
Age (years), *n* (%)			0.583
<65	13 (27.1%)	17 (32.1%)	
≥65	35 (72.9%)	36 (67.9%)	
Sex, *n* (%)			0.625
Female	33 (68.8%)	34 (64.2%)	
Male	15 (31.2%)	19 (35.8%)	
Clinical stage, *n* (%)			0.083
III	30 (62.5%)	24 (45.3%)	
IV	18 (37.5%)	29 (54.7%)	
Location, *n* (%)			0.200
Proximal GC	15 (31.2%)	18 (34%)	
Body GC	23 (47.9%)	17 (32.1%)	
Distal GC	10 (20.8%)	18 (34%)	
CA19-9 (U/mL), *n* (%)			0.956
<37	22 (45.8%)	24 (45.3%)	
≥37	26 (54.2%)	29 (54.7%)	
CEA (ng/mL), *n* (%)			0.091
<5	28 (58.3%)	22 (41.5%)	
≥5	20 (41.7%)	31 (58.5%)	
Smoking history, *n* (%)			0.452
No	38 (79.2%)	45 (84.9%)	
Yes	10 (20.8%)	8 (15.1%)	
Drinking history, *n* (%)			0.424
No	41 (85.4%)	48 (90.6%)	
Yes	7 (14.6%)	5 (9.4%)	
Diabetes history, *n* (%)			0.283
No	38 (79.2%)	37 (69.8%)	
Yes	10 (20.8%)	16 (30.2%)	
History of hypertension, *n* (%)			0.658
No	38 (79.2%)	40 (75.5%)	
Yes	10 (20.8%)	13 (24.5%)	
BMI, mean ± SD	21.89 ± 3.21	21.90 ± 2.53	0.986
HCT, mean ± SD	34.44 ± 4.88	33.66 ± 5.42	0.454

GC, gastric cancer; CA19-9, carbohydrate antigen 19-9; CEA, carcinoembryonic antigen; HCT, hematocrit; BMI, body mass index; mean ± SD, mean ± standard deviation.

### Survival analysis

**Figure 2:**
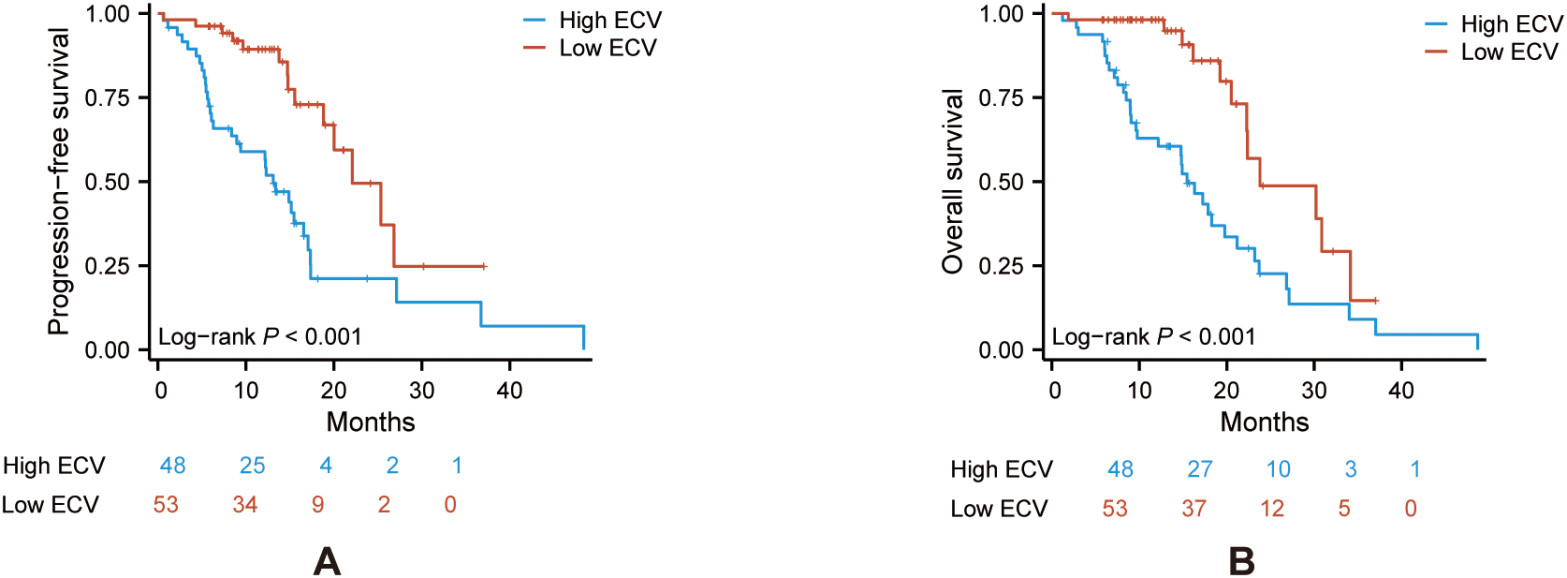
Kaplan–Meier curve of PFS **(A)** and OS **(B)** in the ECV-lower group (red) and ECV-higher group (blue). Analyses were conducted using log-rank tests. ECV, extracellular volume fraction; PFS, progression-free survival; OS, overall survival.

Kaplan–Meier survival curves of PFS and OS were conducted between the two groups. The log-rank tests indicated that the ECV-higher group had a shorter PFS (*P* < 0.001) and OS (*P* < 0.001) than the ECV-lower group (**Figures 2A, B**).

### Cox regression analysis and subgroup analysis

Univariate analysis showed that high ECV significantly predicted a shorter progression-free survival (PFS: HR = 3.194, 95% CI: 1.702–5.993, *P* < 0.001), and clinical stage IV was associated with reduced PFS (HR = 3.078, 95% CI: 1.660–5.707, *P* < 0.001). For OS, high ECV and clinical stage IV showed a stronger prognostic value (HR = 3.220, 95% CI: 1.665–6.230, *P* < 0.001; HR = 3.501, 95% CI: 1.872–6.549, *P* < 0.001).

Multivariate analysis showed that high ECV independently predicted shorter PFS (HR = 2.716, 95% CI: 1.432–5.152, *P* = 0.002) and OS (HR = 2.593, 95% CI: 1.322–5.084, *P* = 0.006). Similarly, clinical stage IV remained significantly linked to both shorter PFS (HR = 2.573, 95% CI: 1.377–4.808, *P* = 0.003) and poorer OS (HR = 2.881, 95% CI: 1.524–5.448, *P* = 0.001) (**Tables 2**, **3**).

**Table 2:** Univariate and multivariate Cox proportional hazards analyses for PFS.

Characteristics	Total (*N*)	Univariate analysis		Multivariate analysis	
		Hazard ratio (95% CI)	*P*-value	Hazard ratio (95% CI)	*P*-value
Sex	101				
Female	34	Reference			
Male	67	1.084 (0.587–2.001)	0.797		
Age	101				
<65 years	71	Reference			
≥65 years	30	1.297 (0.644–2.614)	0.467		
Clinical stage	101				
III	47	Reference		Reference	
IV	54	3.078 (1.660–5.707)	<0.001	2.573 (1.377–4.808)	0.003
Location	101				
Proximal GC	33	Reference			
Body GC	40	1.617 (0.804–3.252)	0.178		
Distal GC	28	1.260 (0.578–2.748)	0.561		
CA19-9	101				
<37 (U/mL)	55	Reference			
≥37 (U/mL)	46	1.273 (0.718–2.258)	0.409		
CEA	101				
<5 (ng/mL)	50	Reference			
≥5 (ng/mL)	51	1.225 (0.677–2.216)	0.503		
Smoking history	101				
No	83	Reference			
Yes	18	1.372 (0.674–2.796)	0.383		
Drinking history	101				
No	89	Reference			
Yes	12	1.366 (0.573–3.257)	0.481		
Diabetes history	101				
No	75	Reference			
Yes	26	1.319 (0.620–2.808)	0.472		
History of hypertension	101				
No	78	Reference			
Yes	23	0.508 (0.229–1.128)	0.096		
ECV group	101				
Low	53	Reference		Reference	
High	48	3.194 (1.702–5.993)	<0.001	2.716 (1.432–5.152)	0.002

PFS, progression-free survival; CI, confidence interval; GC, gastric cancer; CA19-9, carbohydrate antigen 19-9; CEA, carcinoembryonic antigen; ECV, extracellular volume fraction.

**Table 3:** Univariate and multivariate Cox proportional hazards analyses for OS.

Characteristics	Total (*N*)	Univariate analysis		Multivariate analysis	
		Hazard ratio (95% CI)	*P*-value	Hazard ratio (95% CI)	*P*-value
Sex	101				
Female	34	Reference			
Male	67	1.211 (0.642–2.285)	0.554		
Age	101				
<65 years	71	Reference			
≥65 years	30	1.429 (0.726–2.814)	0.301		
Clinical stage	101				
III	47	Reference		Reference	
IV	54	3.501 (1.872–6.549)	<0.001	2.881 (1.524–5.448)	0.001
Location	101				
Proximal GC	33	Reference			
Body GC	40	1.911 (0.941–3.883)	0.073		
Distal GC	28	1.612 (0.736–3.535)	0.233		
CA19-9	101				
<37 (U/mL)	55	Reference			
≥37 (U/mL)	46	1.356 (0.757–2.429)	0.305		
CEA	101				
<5 (ng/mL)	50	Reference			
≥5 (ng/mL)	51	0.996 (0.551–1.801)	0.991		
Smoking history	101				
No	83	Reference			
Yes	18	1.548 (0.782–3.066)	0.210		
Drinking history	101				
No	89	Reference			
Yes	12	1.551 (0.643–3.739)	0.328		
Diabetes history	101				
No	75	Reference			
Yes	26	1.680 (0.733–3.851)	0.220		
History of hypertension	101				
No	78	Reference			
Yes	23	0.678 (0.322–1.428)	0.307		
ECV group	101				
Low	53	Reference		Reference	
High	48	3.220 (1.665–6.230)	<0.001	2.593 (1.322–5.084)	0.006

OS, overall survival; CI, confidence interval; GC, gastric cancer; CA19-9, carbohydrate antigen 19-9; CEA, carcinoembryonic antigen; ECV, extracellular volume fraction.

**Figure 3:**
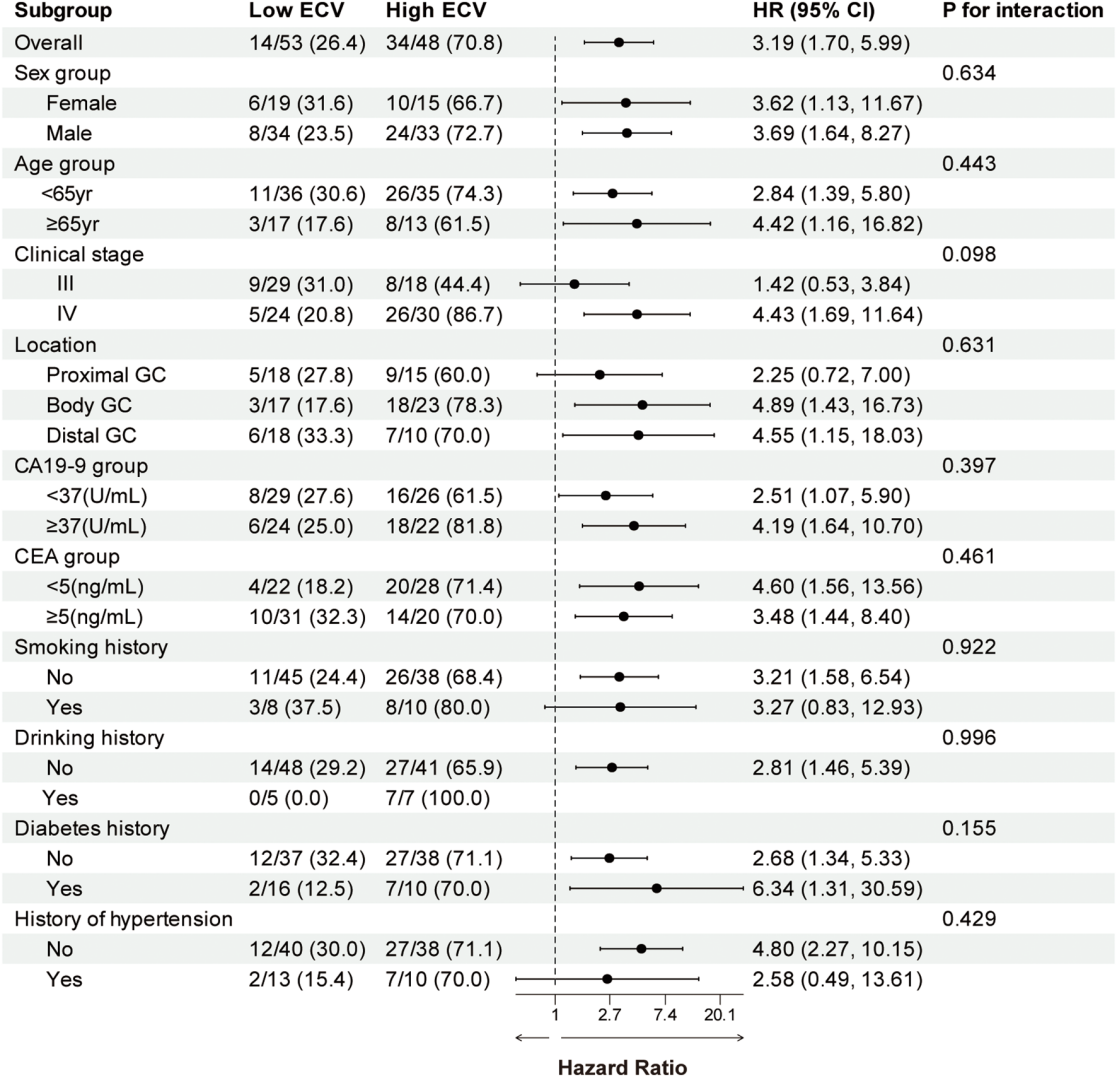
Forest plot of subgroup analysis in progression-free survival between the ECV-lower group and ECV-higher group. The dashed line indicates a hazard ratio of 1. ECV, extracellular volume fraction; HR, hazard ratio; CI, confidence interval; GC, gastric cancer; CA19-9, carbohydrate antigen 19-9; CEA, carcinoembryonic antigen.

**Figure 4:**
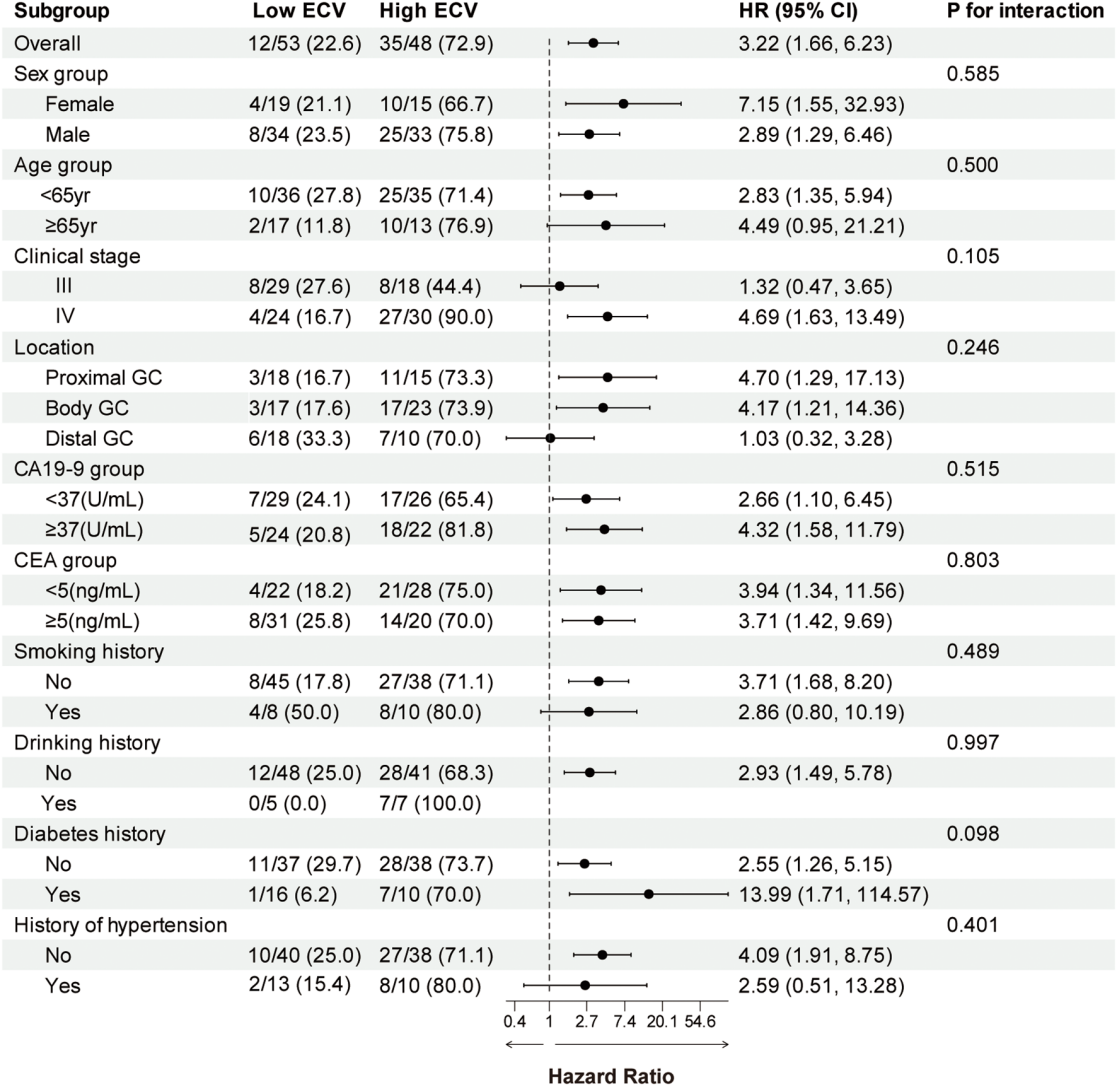
Forest plot of subgroup analysis in overall survival between the ECV-lower group and ECV-higher group. The dashed line indicates a hazard ratio of 1. ECV, extracellular volume fraction; HR, hazard ratio; CI, confidence interval; GC, gastric cancer; CA19-9, carbohydrate antigen 19-9; CEA, carcinoembryonic antigen.

We performed a subgroup analysis of patients based on baseline characteristics and observed relatively consistent results for PFS and OS, and the hazard ratios for each subgroup were derived from the univariate Cox model. Forest plots revealed a uniformly increased risk in high-ECV patients across all subgroups, both in PFS and OS (**Figures 3**, **4**). The association between high ECV and worse survival was consistent within both stage III and stage IV subgroups. No significant heterogeneity of ECV effect was observed (all *P* for interaction >0.05), supporting the robustness of ECV as a universal predictor.

### Combination of multiple indicators for predicting disease progression and survival outcomes

**Figure 5:**
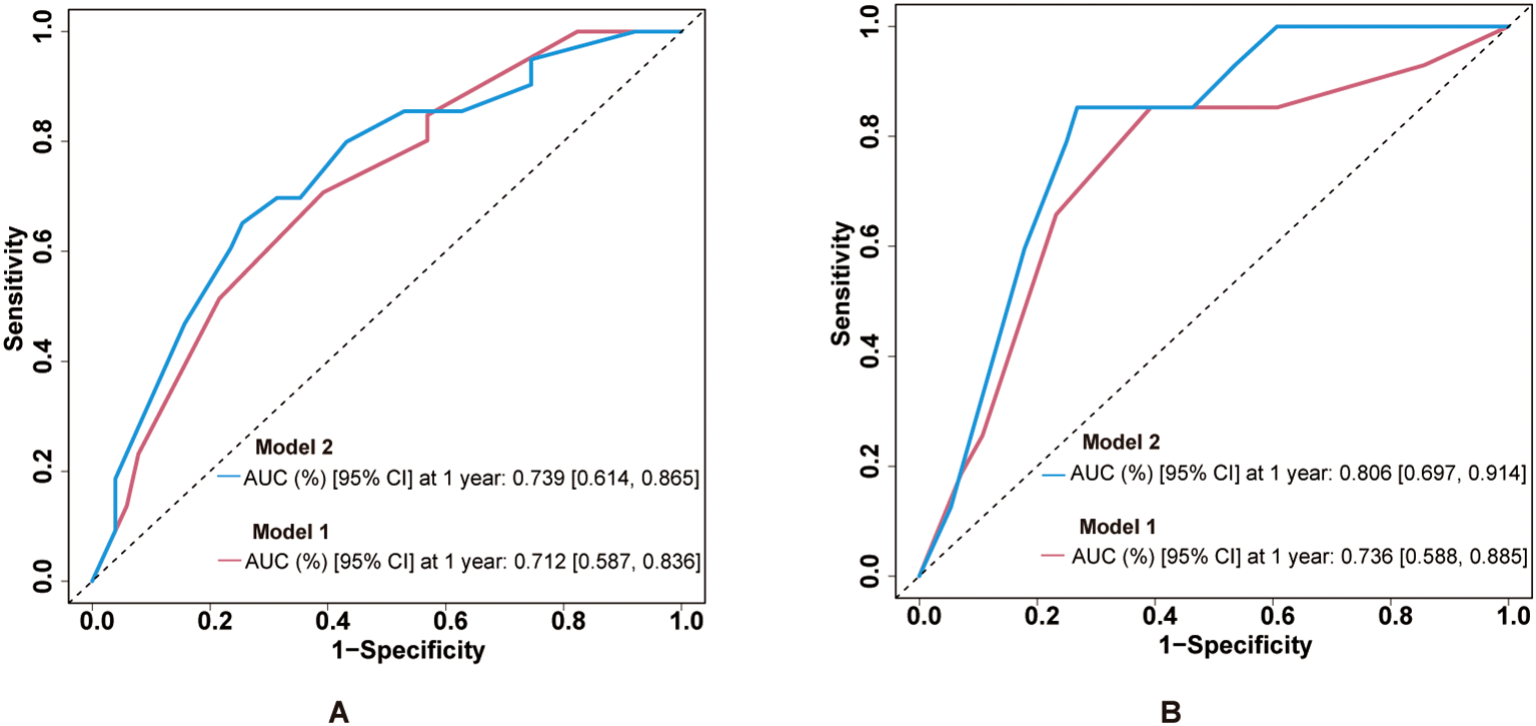
Predictive performance of ECV-integrated models. **(A)** ROC curves for PFS prediction comparing model 1 and model 2. AUC values are shown in the legend. **(B)** ROC curves for OS prediction with the same model comparisons. ECV, extracellular volume fraction; ROC, receiver operating characteristic; AUC, area under the curve; PFS, progression-free survival; OS, overall survival.

ROC curves demonstrated the predictive ability at 1-year survival rate of the combination of clinical stage with ECV for PFS and OS. For PFS prediction (**Figure 5A**), the combined model (model 2: clinical stage, age group, sex, and ECV group) demonstrated superior discriminative ability compared to model 1 (clinical stage, age group, and sex), with AUC values of 0.739 (95% CI: 0.614–0.865) versus 0.712 (95% CI: 0.587–0.836). Similarly, in OS prediction (**Figure 5B**), the integration of ECV (model 2) significantly improved predictive accuracy compared to model 1, demonstrated by an increase in AUC from 0.736 (95% CI: 0.588–0.885) to 0.806 (95% CI: 0.697–0.914). To further assess the robustness and internal validity of the prognostic models, we performed bootstrap resampling (*B* = 1,000) to estimate the time-dependent AUCs and their 95% CIs at 1-year follow-up. Internal validation of the ROC curves was performed using bootstrap resampling with 1,000 iterations. The internally validated results consistently confirmed the superior performance of model 2 compared to model 1 (**Supplementary Figures S2**, **S3**). For OS prediction, the 1-year AUC of model 1 was 0.772 (95% CI: 0.636–0.892); for model 2, the 1-year AUC was 0.835 (95% CI: 0.728–0.932). For PFS prediction, the 1-year AUC of model 1 was 0.734 (95% CI: 0.611–0.848); for model 2, the 1-year AUC was 0.774 (95% CI: 0.646–0.886). These findings suggest that the inclusion of ECV enhanced the discriminative ability of the clinical model and that the results remained stable after internal validation using bootstrap methods.

## Discussion

ECV has traditionally been used to assess fibrosis in organs such as the heart, liver, and pancreas (10, 12, 13), but its role in gastric cancer has rarely been explored. In this retrospective study, we were the first to investigate the prognostic value of ECV in gastric cancer patients receiving immunotherapy. Multivariate Cox regression analysis, which adjusted for the influence of clinical stage, confirmed that high CT-ECV was independently associated with worse PFS (HR = 2.716, 95% CI: 1.432–5.152, *P* = 0.002) and OS (HR = 2.593, 95% CI: 1.322–5.084, *P* = 0.006). Moreover, integrating ECV into a clinical-stage-based prognostic model improved its predictive performance. The combined model demonstrated higher area under the ROC curve values (PFS: AUC = 0.739, 95% CI: 0.614–0.865; OS: AUC = 0.806, 95% CI: 0.697–0.914), supporting the added prognostic value of ECV. Internal validation using bootstrap resampling (*B* = 1,000) confirmed the consistency and robustness of the combined model, further reinforcing the credibility of our findings. Collectively, these results suggest that ECV may serve as a noninvasive imaging biomarker, with potential utility in predicting immunotherapy outcomes.

Of the existing biomarkers for gastric cancer immunotherapy, such as PD-L1 expression, MSI, tumor mutational burden (TMB), EBV status, liquid biopsy, and emerging multi-omics approaches, many have significantly improved the precision of predicting immunotherapy efficacy. However, the clinical utility of these biomarkers is constrained by several factors, including spatial and temporal heterogeneity, sampling bias, invasiveness of tissue acquisition, dynamic changes in PD-L1 expression, the high cost and technical complexity associated with genomic profiling like TMB, as well as the poor interpretability and inherent complexity of radiomics models (27). Even emerging non-invasive methods such as liquid biopsy face challenges related to sensitivity, specificity, and the potential for false-positive results (28). In contrast, the CT-ECV framework evaluated in this study utilizes standard contrast-enhanced CT scans, making it a non-invasive, readily accessible, and cost-effective tool. More importantly, ECV provides unique biological insight by directly quantifying a fundamental and functionally critical aspect of the tumor microenvironment (TME)—the fibrotic extracellular matrix (ECM). As our results and the discussed mechanisms suggest, a high ECV likely reflects a stroma-rich, immunosuppressive TME characterized by dense physical barriers that impede immune cell infiltration and drug delivery (29). Therefore, although ECV, like any novel biomarker, requires further validation in larger cohorts, it is not intended to replace existing markers but rather to serve as a highly practical and complementary biomarker.

Increased stiffness of the ECM is considered a key factor in promoting an immunosuppressive tumor microenvironment. It can stimulate M2 macrophage polarization through mechanotransduction pathways and induce the release of immunosuppressive cytokines. Additionally, ECM stiffening contributes to tissue hypoxia and activates the HIF-α signaling pathway, collectively leading to an immunosuppressive and T-cell-exhausted microenvironment. Furthermore, the dense structure of the ECM not only forms a physical barrier that limits immune cell infiltration into the tumor but also exerts mechanical tension that activates mechanosensitive signaling pathways. As a result, T-cell migration is restricted, and anti-PD-1 antibodies may have difficulty penetrating into the tumor core, ultimately compromising the efficacy of immunotherapy (30–32). The tumor microenvironment, particularly the stromal component, plays a crucial part in GC progression. Previous studies have demonstrated that tumor-associated stroma plays an active role in tumor invasion and metastasis and that GC with high stromal content are associated with worse prognosis compared to those with low stromal content (33–35). Moreover, it has been reported that collagen density modulates the activity of tumor-infiltrating T cells, with high collagen density leading to reduced T-cell proliferation and impaired cytotoxic function, ultimately promoting immune escape by tumor cells (36). Consistent with previous findings in postoperative gastric cancer, elevated CT-derived ECV has been shown to reflect stromal-rich tumor microenvironments that may facilitate tumor progression and resistance (23). Our study extends this concept into the immunotherapy setting. ECV, a noninvasive imaging biomarker, serves as an indirect indicator of the amount and spatial distribution of ECM within the tumor tissue, which may explain the poorer prognosis observed in patients with high ECV undergoing immunotherapy (9). Interestingly, in some cancers such as pancreatic adenocarcinoma, higher ECV has been associated with better drug distribution and longer survival (16, 37). These divergent associations may be explained by fundamental differences in the biological nature of the tumor stroma across cancer types coupled with the distinct mechanisms of action of different therapeutic regimens. We propose that this paradox hinges on a fundamental question: does the treatment strategy actively attack the stromal barrier, or must it bypass it? In pancreatic cancer, cornerstone chemotherapies (e.g., nab-paclitaxel plus gemcitabine) function as stroma-depleting agents, physically breaking down the fibrotic barrier (38). Here a high baseline ECV may simply mark a larger, more susceptible target for chemotherapy regimen. However, in the context of immunotherapy, this phenomenon does not necessarily translate into improved therapeutic outcomes. PD-1 inhibitors do not dismantle the barrier but require T-cells to traverse it. Thus, an elevated ECV may indicate a denser stromal architecture, restricted T-cell migration, and enhanced immunosuppression, which could counteract the potential benefits of increased drug dispersion. This comparison highlights that ECV quantifies a dynamic interface whose impact is determined by the therapy applied. These findings suggest that further mechanistic studies and external validation are warranted in diverse cancer types and immune phenotypes to better understand the complex relationship between ECV and immunotherapy response.

This study has several limitations. First, being a retrospective single-center study, the findings may be subject to selection bias and limited generalizability despite the use of strict inclusion criteria. Second, the relatively small sample size restricted the statistical power of some subgroup analyses, although we performed internal validation using bootstrap methods to enhance reliability. Third, ECV was measured based on conventional single-energy CT rather than dual-energy or spectral CT, which may introduce variability; however, standardized imaging protocols and ROI selection helped minimize this issue. Lastly, external validation in independent cohorts was not conducted, and prospective multicenter studies are warranted to confirm the generalizability and clinical applicability of our model. In conclusion, CT-derived ECV may serve as an independent prognostic factor in gastric cancer patients treated with immunotherapy. As a noninvasive imaging biomarker, ECV holds promise for risk stratification and therapeutic decision-making in this patient population and warrants further validation in clinical practice.

## Conclusion

This retrospective cohort study of gastric cancer patients undergoing immunotherapy demonstrated that contrast-enhanced CT-derived ECV may serve as an independent predictor for both PFS and OS patients with low ECV who exhibited improved PFS and OS compared to those with high ECV.

## Data Availability

The raw data supporting the conclusions of this article will be made available by the authors, without undue reservation.
