# Supplementary material for: Prediction of long-term survival in gastric cancer patients after immunotherapy based on CT-derived extracellular volume fraction
Source: Front Oncol. 2025 Nov 28;15:1698065. doi: 10.3389/fonc.2025.1698065 (PMC12698398; doi:10.3389/fonc.2025.1698065)
Supplement: Supplementary file 1 [file DataSheet1.pdf]

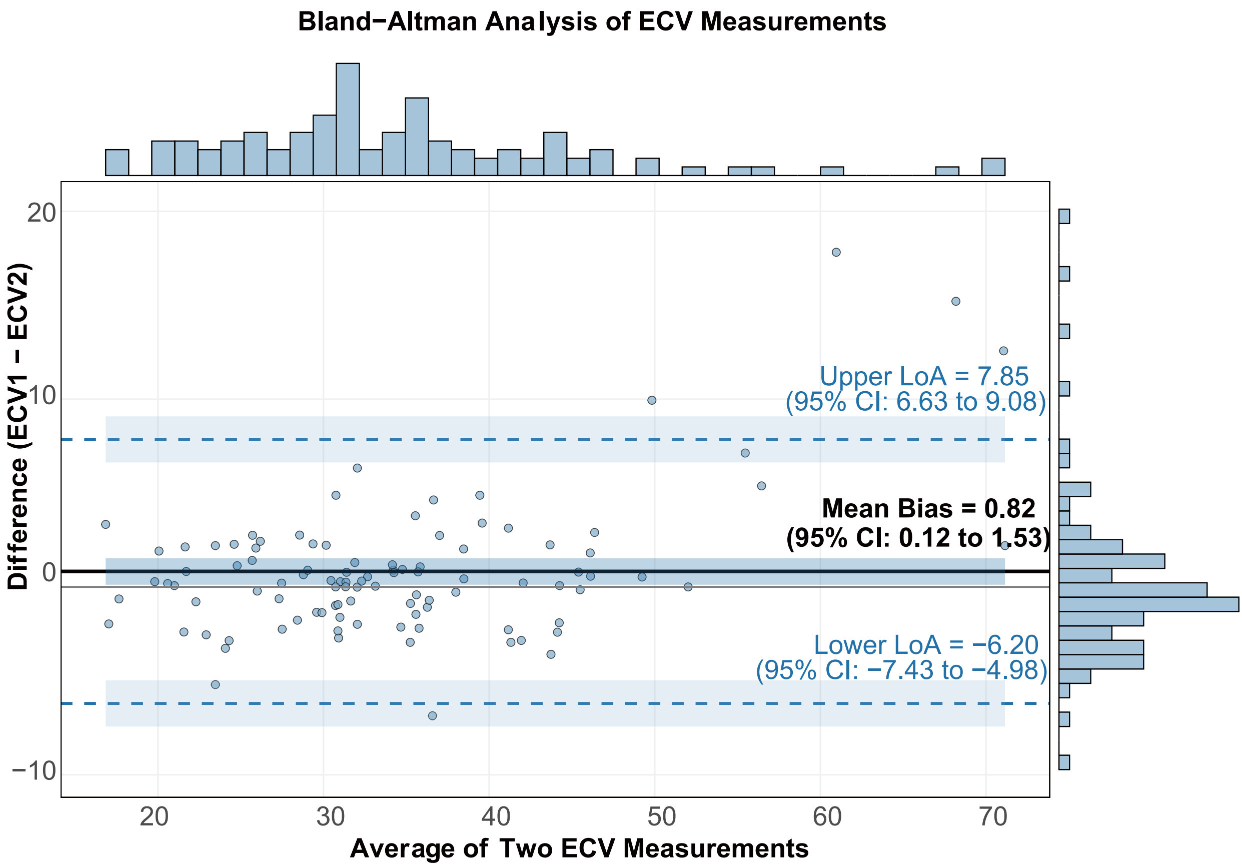

Supplementary Figure 1. Bland-Altman analysis of interobserver agreement in CT-ECV measurements. The difference between the two radiologists' ECV calculations is plotted against their mean values. Blue points represent individual paired measurements from two radiologists. The solid black line indicates the mean bias, while dashed blue lines represent 95% LoA. Dark blue band shows the 95% CI for the mean difference, and light blue bands indicate 95% CIs for the LoA. A blue marginal histogram is appended to the Bland-Altman analysis chart. The top histogram shows the distribution of mean ECV measurements, and the right histogram shows the distribution of measurement differences. ECV, extracellular volume fraction; LoA, limits of agreement; CI, confidence interval.
