# Supplementary material for: Prediction of long-term survival in gastric cancer patients after immunotherapy based on CT-derived extracellular volume fraction
Source: Front Oncol. 2025 Nov 28;15:1698065. doi: 10.3389/fonc.2025.1698065 (PMC12698398; doi:10.3389/fonc.2025.1698065)
Supplement: Supplementary file 2 [file DataSheet2.pdf]

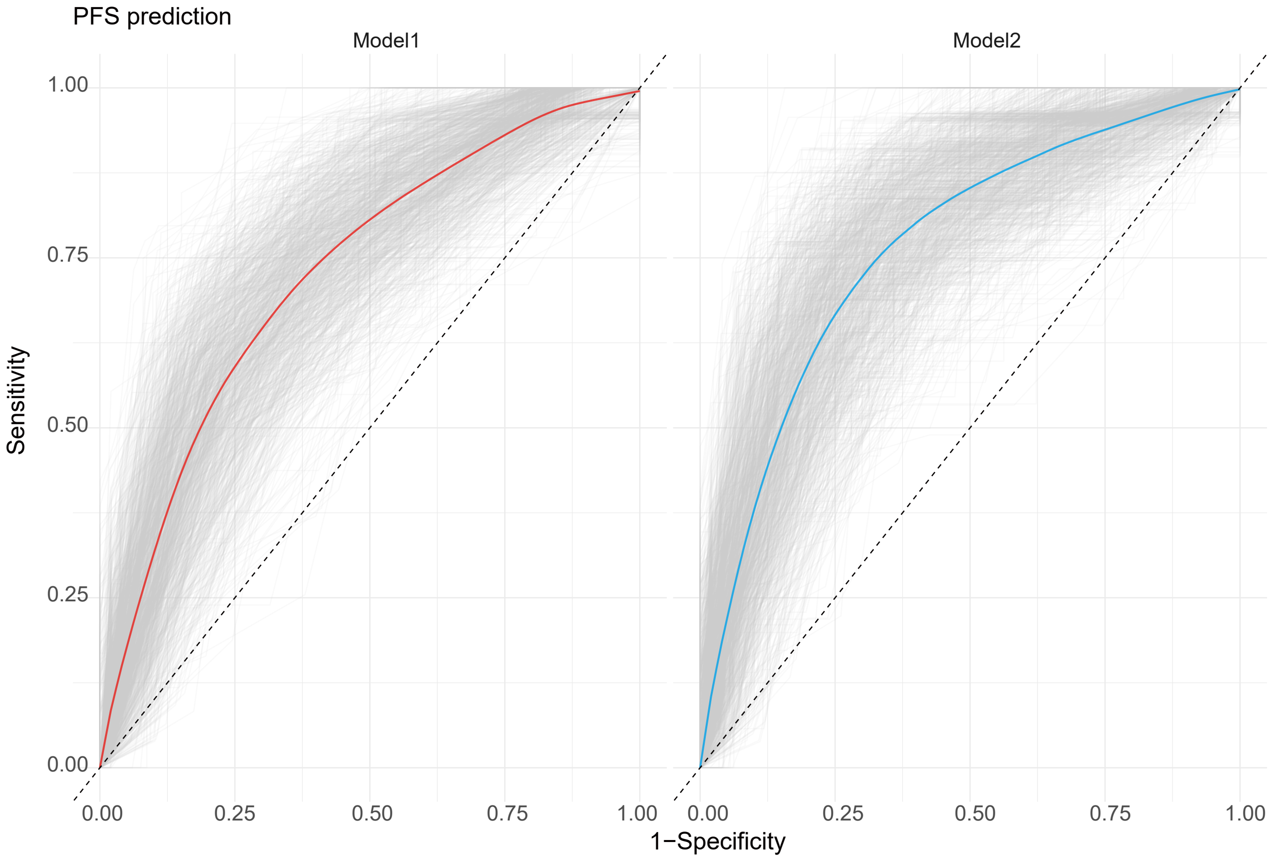

Supplementary Figure 2. Bootstrap-validated ROC curves after 1,000 resampling iterations in PFS. The shaded areas represent 95% CI bands. Model 1 (red): Clinical stage + age + sex. Model 2 (blue): Model 1 + ECV stratification. The dashed line indicates reference (AUC=0.50). PFS, progression-free survival; ECV, extracellular volume fraction.
